# Sodium accumulation in breast cancer predicts malignancy and treatment response

**DOI:** 10.1038/s41416-022-01802-w

**Published:** 2022-04-25

**Authors:** Andrew D. James, Theresa K. Leslie, Joshua D. Kaggie, Laura Wiggins, Lewis Patten, John Murphy O’Duinn, Swen Langer, Marie-Christine Labarthe, Frank Riemer, Gabrielle Baxter, Mary A. McLean, Fiona J. Gilbert, Aneurin J. Kennerley, William J. Brackenbury

**Affiliations:** 1 Department of Biology, University of York, York, UK; 2 York Biomedical Research Institute, University of York, York, UK; 3 Department of Radiology & NIHR Cambridge Biomedical Research Centre, University of Cambridge, Cambridge, UK; 4 Department of Mathematics, University of York, York, UK; 5 Department of Chemistry, University of York, York, UK; 6 Bioscience Technology Facility, Department of Biology, University of York, York, UK; 7 Department of Physics, University of York, York, UK; 8 Mohn Medical Imaging and Visualization Centre, Haukeland University Hospital Bergen, Bergen, Norway

**Keywords:** Breast cancer, Cancer imaging

## Abstract

**Background:**

Breast cancer remains a leading cause of death in women and novel imaging biomarkers are urgently required. Here, we demonstrate the diagnostic and treatment-monitoring potential of non-invasive sodium (^23^Na) MRI in preclinical models of breast cancer.

**Methods:**

Female *Rag2*^−/−^ *Il2rg*^−/−^ and Balb/c mice bearing orthotopic breast tumours (MDA-MB-231, EMT6 and 4T1) underwent MRI as part of a randomised, controlled, interventional study. Tumour biology was probed using ex vivo fluorescence microscopy and electrophysiology.

**Results:**

^23^Na MRI revealed elevated sodium concentration ([Na^+^]) in tumours vs non-tumour regions. Complementary proton-based diffusion-weighted imaging (DWI) linked elevated tumour [Na^+^] to increased cellularity. Combining ^23^Na MRI and DWI measurements enabled superior classification accuracy of tumour vs non-tumour regions compared with either parameter alone. Ex vivo assessment of isolated tumour slices confirmed elevated intracellular [Na^+^] ([Na^+^]_i_); extracellular [Na^+^] ([Na^+^]_e_) remained unchanged. Treatment with specific inward Na^+^ conductance inhibitors (cariporide, eslicarbazepine acetate) did not affect tumour [Na^+^]. Nonetheless, effective treatment with docetaxel reduced tumour [Na^+^], whereas DWI measures were unchanged.

**Conclusions:**

Orthotopic breast cancer models exhibit elevated tumour [Na^+^] that is driven by aberrantly elevated [Na^+^]_i_. Moreover, ^23^Na MRI enhances the diagnostic capability of DWI and represents a novel, non-invasive biomarker of treatment response with superior sensitivity compared to DWI alone.

## Background

Metastatic breast cancer represents the leading cause of cancer-related death in women worldwide [1], with the triple-negative subtype associated with particularly poor prognosis [2]. Current standard care pathways involve mammography, ultrasound, magnetic resonance imaging (MRI) and biopsy for diagnostics [3]. The inclusion of additional MRI modalities in care pathways can improve outcomes via early diagnosis, structural and morphological assessment of tumours and monitoring of therapy response [4]. Additional non-invasive MRI biomarkers could therefore contribute a significant clinical improvement.

Recent MR advances have aimed to address the unmet clinical need for better patient stratification. Dynamic contrast-enhanced (DCE) ^1^H MRI, metabolic imaging (PET/MR) and hyperpolarized pyruvate (^13^C labelled) approaches provide functional information about neoangiogenesis [4] and tumour metabolic activity [5], respectively. Unfortunately, these three approaches are invasive and often limited by safety concerns (e.g. gadolinium-based contrast agent accumulation) and/or requirements for complex equipment [6]. As such, non-invasive, proton-based diffusion-weighted imaging (^1^H DWI), which determines the local restriction of water within a tissue through quantification of the apparent diffusion coefficient (ADC), is now commonly used clinically for detecting malignant breast lesions [4, 7]. Low ADC values are correlated with malignancy and correspond with small extracellular volume fractions and high cellularity [8–10]. Furthermore, DWI demonstrates improved predictive value over conventional DCE MRI [11].

Moving away from conventional proton-based imaging, previous studies indicate that non-invasive ^23^Na MRI of tumour sodium concentration ([Na^+^]) could be a biomarker of malignancy in breast cancer [8, 12, 13]. The receptivity of ^23^Na MRI is orders of magnitude lower than that of ^1^H MRI [14] due to the lower tissue concentration of Na^+^ vs H^+^ and the inherently lower gyromagnetic ratio. However, technological advances (increased magnetic field strength, improved imaging gradients for fast-readout strategies) have enabled ^23^Na imaging within clinically reasonable timeframes [15]. Indeed, clinical evidence indicates that [Na^+^] is elevated within malignant lesions compared with benign lesions [8, 12, 13]; moreover, two prospective clinical studies suggest that response to neoadjuvant chemotherapy correlates with a decrease in tumour [Na^+^] [12, 16]. Intriguingly, low ADC correlates with elevated total tissue [Na^+^] in malignant lesions [8], suggesting a link between high cell density and tumour [Na^+^]. As a result, ^23^Na MRI may have utility for breast cancer diagnosis, risk-stratification and monitoring treatment response. However, elevated breast tumour [Na^+^] remains poorly characterised, and urgent supporting data are required for this approach to gain clinical footing and to understand the underlying pathophysiology.

Identifying the location of tumour Na^+^ accumulation is critically important for determining its pathophysiological impact. Increases in total tumour [Na^+^] could reflect changes in intracellular sodium ([Na^+^]_i_), extracellular sodium ([Na^+^]_e_), or an increase in the extracellular compartment volume [17]. Altered [Na^+^]_i_ or [Na^+^]_e_ would have dramatic functional implications for tumour biology; elevations in [Na^+^]_e_ are proinflammatory, inhibit immune cell function [18], and promote resistance to chemotherapy [19], whereas elevations in [Na^+^]_i_ (reported in cultured cancer cells [20, 21] and in ex vivo tumour samples [22]) could reflect alterations in processes dependent on the inward Na^+^ gradient [17, 23]. Regarding the latter, many characteristics of invasive tumours (e.g. cell migration, acidification of the tumour microenvironment) are regulated by inward Na^+^ transport [20, 24]. The Na^+^ dependent Na^+^/H^+^ exchanger (NHE1) regulates tumour pH and metastasis [25]; NHE1 blockade with cariporide inhibits tumour growth [26] and sensitises cancer cells to chemotherapy [27]. Moreover, voltage-gated Na^+^ channels (VGSCs) expressed in breast cancer cells pass a persistent inward Na^+^ current, regulate metastatic cell behaviour and are inhibited using existing antiepileptic medications [20, 23, 28–31]. Determining the precise location and mechanism of tumour Na^+^ accumulation could therefore reveal novel druggable targets.

In this study, we used mouse models of breast cancer to assess whether the effects of chemotherapy on breast tumour [Na^+^] are reproducible beyond early clinical observations [12, 16] and investigated whether specifically targeting Na^+^ conductance mechanisms affects tumour [Na^+^]. To date, these questions have not been addressed in preclinical in vivo models of breast cancer. Our results indicate that multiple orthotopic tumour models (MDA-MB-231, EMT6, 4T1) exhibit elevated tumour [Na^+^], thus recapitulating the clinical picture. We provide ex vivo evidence that the elevated tumour [Na^+^] reflects high [Na^+^]_i_ rather than [Na^+^]_e_ and show that effective treatment with the chemotherapeutic drug docetaxel reduces tumour [Na^+^] but is not reflected by MR changes in ADC. Importantly, we show that while tumour [Na^+^] has a similar predictive capacity to ADC for classifying malignant regions, the combination of these parameters provides superior prediction accuracy. These findings indicate that ^23^Na MRI has utility as a non-invasive biomarker for both malignant disease and treatment response, and position aberrant intracellular Na^+^ handling as a critical feature of malignant breast tumours that may represent an important therapeutic target.

## Methods

### Cell culture

All cells were cultured in a humidified atmosphere of air/CO_2_ (95:5%) at 37 °C and routinely tested for mycoplasma using the DAPI method. Cell line sources, media and supplements are described in Supplementary Methods.

### Orthotopic breast tumour model

*Rag2*^*−/*−^ *Il2rg*^−/−^ (bred in-house, Balb/c background strain) or Balb/c (Charles River Laboratories, UK; acclimatised for 2 weeks) mice were housed in individually ventilated cages with enrichment (3–5 mice per cage) in temperature-controlled rooms with access to water and food ad libitum. At >6 weeks of age, female mice were anaesthetised (2% isoflurane in oxygen (2 l/min)) and 5 × 10^5^ MDA-MB-231 (*Rag2*^*−*/−^ *Il2rg*^−/−^), 1 × 10^5^ EMT6 or 1 × 10^5^ 4T1 (BALB/c) cells (suspended in Matrigel, 50% v/v in saline, 50 µl of volume) were injected into the left inguinal mammary fat pad. Animal weight, condition and tumour growth (calliper measurement) were monitored daily. Where possible, tumour volume was calculated from weekly multislice ^1^H scans; otherwise, tumour volumes were calculated from calliper measurements (modified ellipsoidal formula, volume = 1/2(length × width^2^) [32]. Mice were euthanized at <5 weeks after cell implantation or at 15-mm tumour diameter and tumours isolated.

### Drug treatment interventional studies

Tumour-bearing mice were randomised (blocked by cage) to receive either vehicle or drug treatment from day 7 post tumour cell implantation (docetaxel 10 mg/kg in 1:1:20 ethanol:Tween 20:5% glucose in H_2_O once weekly i.p., total 18 mice across six cages, docetaxel *n* = 11, vehicle, *n* = 7; cariporide 3 mg/kg in 50:1 PBS:DMSO once daily i.p., total 10 mice across 3 cages, cariporide *n* = 5, vehicle, *n* = 5; ESL, 175 or 200 mg/kg in 0.5% methylcellulose suspension once daily p.o., total 7 mice across 2 cages, ESL *n* = 4, vehicle, *n* = 3). No power calculation for sample sizes was performed as there is no prior study on which this could be based. Drug stocks were prepared as follows: docetaxel, ethanol (final dilution 4% v/v); cariporide, DMSO (final dilution 2%); ESL was directly suspended in the vehicle.

### MRI

All experiments were performed on a 7T preclinical MRI system (Biospec 70/30 USR AVANCE III, Bruker Biospin) with a 12 channel RT-shim system (B-S30) and preinstalled 660mT/m imaging gradient set (BGA-12s, Bruker). Data were captured using Paravision 6.0.1 software. Details of the MRI transceive coils used and all acquisition sequence settings for ^23^Na MRI and ^1^H DWI are described in Supplementary Methods.

All live-animal experiments were performed with a NaCl phantom (50 mM, 1.5-ml centrifuge tube, inner diameter 8.2 mm) to enable normalisation of signal between animals. Imaging was performed at 2, 3 and 4 weeks (MDA-MB-231 xenografts) or at 7 days (4T1 and EMT6 xenografts) post implant. Animals without palpable tumours were excluded. Mice were anaesthetised (2% isoflurane in 2 l/min O_2_) and breathing monitored using a pressure-sensitive pad (MR-Compatible Model 1025 Monitoring and Gating System, SAII, NY, USA) throughout. Body temperature was maintained at 37 °C using a water-heated bed.

### Ex vivo SBFI fluorescence imaging

Following euthanasia (week-4 MRI timepoint), tumours were isolated and sliced (200 µm) in chilled phosphate-buffered saline using a 5100 MZ vibratome (Campden Instruments Ltd). Tumour slices were loaded with SBFI-AM (10 µM) in HEPES-buffered physiological saline solution (HEPES-PSS: 144 mM NaCl, 5.4 mM KCl, 1 mM MgCl_2_, 1.28 mM CaCl_2_, 5 mM HEPES and 5.6 mM glucose, pH 7.2) with 0.08% Pluronic F-127 for 2 h at 37 °C, then rinsed and incubated in fresh HEPES-PSS for 40 minutes. Microscopy equipment used for SBFI fluorescence imaging is described in Supplementary Methods.

Na^+^-free HEPES-PSS was prepared by replacing NaCl with equimolar *N*-methyl-d-glucamine (NMDG, 144 mM). HEPES-PSS for calibrating [Na^+^]_i_ was prepared containing 10, 20 and 50 mM NaCl, with additional KCl added in place of omitted NaCl (149.4 mM NaCl + KCl, 1 mM MgCl_2_, 1.28 mM CaCl_2_, 5 mM HEPES, 5.6 mM glucose, adjusted to pH 7.2 with KOH). Following baseline measurement, slices were perfused with 10 mM [Na^+^]_e_. Na^+^ ionophores (gramicidin, 3 µM; monensin, 10 µM) were applied to equilibrate [Na^+^]_i_ and [Na^+^]_e_ and a Na^+^/K^+^ ATPase inhibitor (ouabain, 1 mM) was applied to inhibit Na^+^ efflux. [Na^+^]_e_ was sequentially changed to 20 mM and 50 mM [33]. [Na^+^]_i_ was calibrated in situ to control for differences between cells using the final three acquisition frames for each perfusion phase.

### Ion-selective microelectrodes

Methods for producing Na^+^ ion-selective microelectrodes and use of associated recording hardware are described in Supplementary Methods. Isolated tumours were sliced (500 µm) as described for ex vivo SBFI fluorescence imaging and then maintained at 30 °C at an interface between HEPES-PSS perfusion and humidified air. Electrodes were calibrated with HEPES-PSS of variable [Na^+^] (48, 96, 144 and 192 mM, no replacement ion) prior to and after recording. Sensitivity (mV/mM) typically changed by no more than 5% over the duration of an experiment. Recordings were taken from the top surface of tumour slices and from different locations within each slice. The junction potential offset between the bath and calibration tube was subtracted from the recorded voltage. All recordings were made within 90 min of euthanasia.

### Inductively coupled plasma mass spectrometry (ICP-MS)

Total tissue [Na^+^] was determined by inductively coupled plasma mass spectrometry (ICP-MS, 7700x, Agilent). ICP-MS analysis and quantification of tissue [Na^+^] are described in Supplementary Methods.

### LC-MS analysis of plasma and tumour [licarbazepine]

Tissue samples from xenograft tumours were snap-frozen and homogenised in chilled 0.1 M phosphate buffer (pH 5, 1:4 w/v). Lysate preparation and analysis by LC-MS are described in Supplementary Methods. Tumour and plasma [licarbazepine] are reported as ng licarbazepine per mg tumour (ng/mg) and ng/µl, respectively.

### Immunohistochemistry

Cryoprotected tumour samples (sequential overnight incubations in 4% paraformaldehyde, 10% sucrose, 30% sucrose) were embedded and frozen in optimal cutting temperature embedding compound (VWR International). Samples were sectioned (12 µm, LEICA CM1950 cryostat) onto SuperFrost Plus™ Adhesion slides (Thermo Scientific, Waltham, MA) and immunohistochemistry performed using a rabbit anti-Ki67 primary antibody (1:500; Abcam AB15580) with an Alexa-568-conjugated goat anti-rabbit secondary antibody (1:500; Invitrogen). Sections were mounted in Prolong Gold with DAPI (Invitrogen) and scanned (Zeiss AxioScan.Z1 slide scanner, ×20 magnification).

### Data analysis

MRI image processing, within the MATLAB R2019b (MathWorks, MA) environment, was performed blinded. Regions of interest (ROI) covering the tumour, phantom and contralateral non-tumour tissue were manually selected using geometry matched, high-resolution ^1^H TurboRARE structural images (see Supplementary Methods). Per subject, mean and maximum ^23^Na signal data from these ROIs were all normalised by the phantom ROI (50 mM NaCl). ROI size was also used to estimate tumour volume. For data acquired using the ^23^Na surface coil, images were corrected for B1 field inhomogeneity prior to processing. Field correction utilised a prior 1/r^2^ correction based upon images from a suitable 1 M NaCl phantom.

DWI data were analysed using RStudio version 1.2.5033 (RStudio Inc., MA). ADC was calculated from the mean ROI signal, S, as ADC = − ln(S/S_0_)/*b*, where *b* corresponds to the calculable degree of diffusion weighting applied (based upon diffusion gradient timings and applied gradient strength—see Supplementary Methods) in s/mm^2^ and S_0_ is the signal intensity with *b* = 0 (also commonly referred to as an A_0_ image).

Classification analysis was completed in RStudio version 1.2.5033. Initial model classification accuracy (maximum vs mean ^23^Na signal) was determined with leave-one-out cross-validation (LOOCV). We followed this approach due to the limited size of the training datasets (Fig. 2e, f). A further independent test set (vehicle group, Fig. 4) was used to compare models trained on maximum ^23^Na signal vs ADC. Principal component analysis was performed using the prcomp() function and “factoextra” package [34]. Linear discriminant analysis (LDA) with LOOCV was performed using the “MASS” package [35]. The receiver-operating characteristic curve was determined using the “ROCR” package [36].

To calibrate [Na^+^]_e_, ISME recordings from standards were fitted to a non-linear regression (Padé (1,1) approximant), and tumour slice recordings were interpolated. Similarly, SBFI fluorescence recordings for each calibration step were fit to non-linear regression (*n* = 3 experiments, one-phase association) and resting [Na^+^]_i_ (baseline fluorescence) interpolated.

Quantification of Ki67-positive nuclei (as a percentage of nuclei count, DAPI) was performed using ZEN 3.2 (Zeiss, Oberkochen, Germany) and ImageJ 1.53c (NIH, public domain) particle count (50 pixels minimum size) with the Classic Watershed method applied [37].

All statistical comparisons were performed using GraphPad Prism 9.3.1 (GraphPad Software, San Diego, CA). Normal data distribution was confirmed using Shapiro–Wilk tests. Statistical comparisons at each independent timepoint were performed with unpaired, two way Student’s *t* tests. Correlations were assessed using Pearson’s *r* tests. Comparisons between tumour models (Fig. 6) were performed using one-way ANOVA with post hoc Tukey test for multiple comparisons.

## Results

### ^23^Na MRI reveals elevated tumour [Na^+^] in a preclinical model of triple-negative breast cancer

**Fig. 1:**
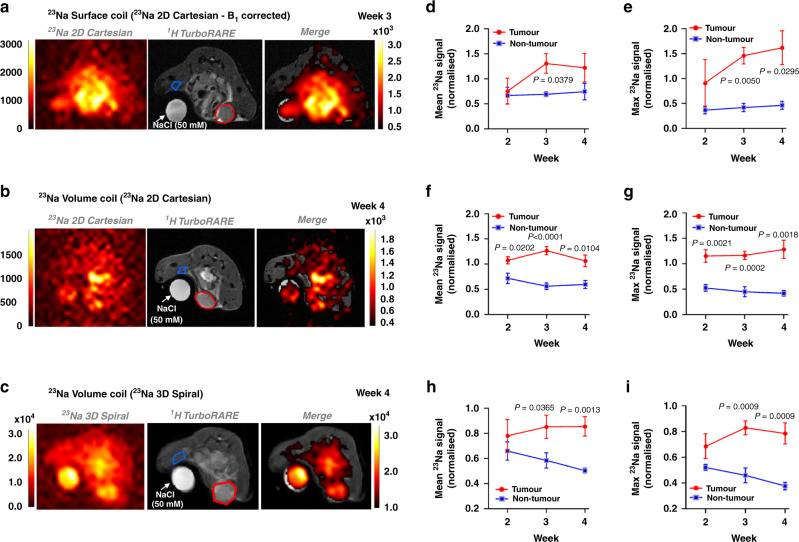
^23^Na MRI reveals elevated [Na^+^] in orthotopic xenograft tumours. ^23^Na imaging was performed in *Rag2*^*−*/−^ *Il2rg*^*−*/−^ mice bearing orthotopic MDA-MB-231 xenograft tumours at 2, 3 and 4 weeks post implantation. Representative images are shown for MRI performed using a bespoke 3 cm ^23^Na surface coil (**a**, ^23^Na 2D gradient-echo cartesian acquisition (2D cartesian)) or a commercial dual-tuned ^1^H/^23^Na volume coil (**b**, ^23^Na 2D cartesian; **c** ^23^Na 3D gradient-echo spiral out acquisition (3D spiral)). Regions of interest (ROIs) are annotated (tumour, red region; non-tumour, blue region). Mean and maximum ^23^Na signals were normalised to a NaCl phantom (50 mM) to provide a concentration measurement. Mean and maximum ^23^Na signals for tumour vs non-tumour regions are shown for the ^23^Na surface coil (**d**, **e**, ^23^Na 2D cartesian, *n* = 3 mice at all timepoints) and dual-tuned ^1^H/^23^Na volume coil (**f**, **g** ^23^Na 2D cartesian. Week 2, *n* = 5; week 3, *n* = 6; week 4, *n* = 5. **h**, **i** ^23^Na 3D spiral. Week 2, *n* = 3; week 3, *n* = 6; week 4, *n* = 6). Data represent group mean ± SEM. *P*-values for significant differences between tumour and non-tumour at each individual timepoint calculated using an unpaired, two-tailed Student’s *t* test. A mixed-effects model indicated that the effect of time was not statistically significant.

Orthotopic MDA-MB-231 xenograft tumours represent a model of highly invasive, triple-negative breast cancer. Across a 4-week longitudinal study, ^23^Na MRI revealed tissue [Na^+^] is significantly elevated within MDA-MB-231 xenografts compared with contralateral non-tumour regions (Fig. 1a–c, tumour growth shown in Supplementary Fig. 1a). ^23^Na signal was linear with [NaCl] (0–100 mM, Supplementary Fig. 1b–e). The observation that [Na^+^] was significantly elevated within tumours was independent of coil setup, readout method and TR. A 3 cm ^23^Na surface coil (B_1_ corrected, ^23^Na 2D gradient-echo Cartesian acquisition [2D Cartesian], Fig. 1a) revealed significantly elevated maximum ^23^Na signal (increased 2.5-fold at week 3, Fig. 1e) and mean ^23^Na signal (increased 0.9-fold at week 3, Fig. 1d) in tumour regions compared with non-tumour regions. A dual-tuned linear ^1^H/^23^Na volume coil (^23^Na 2D Cartesian, Fig. 1b) also detected a significantly higher mean ^23^Na signal (increased 1.3-fold at week 3, Fig. 1f) and maximum ^23^Na signal (increased 1.6-fold at week 3, Fig. 1g) in tumour regions compared with non-tumour regions. ^23^Na 3D gradient-echo spiral out acquisition [3D spiral] with the dual-tuned volume coil (Fig. 1c) also found significant elevations in both mean ^23^Na signal (increased 0.5-fold at week 3, Fig. 1h) and maximum ^23^Na signal (increased 0.8-fold at week 3, Fig. 1i) in tumour regions compared with non-tumour regions. Lower normalised ^23^Na signals from 3D spiral acquisition reflect variable T1 relaxation times between phantom and tissue and a shorter acquisition repetition time (^23^Na 2D Cartesian, TR = 50 ms; ^23^Na 3D spiral, TR = 10 ms). Shorter TR times for the 3D acquisition were required to increase the number of scan repetitions within a set 10-min scanning period. Core temperature was monitored/maintained using pumped warm water. No subject displayed signs of excessive heating due to RF exposure. It is noted that in order to minimise T1 saturation influences, within-group comparisons (across the three different ROIs, phantom/tissue/tumour) use the same coil/sequence parameters over the longitudinal growth measure. Signals are presented normalised to the in-plane phantom ROI (50 mM). Thus, while data may not be quantitative in terms of absolute mM concentrations, normalised changes over time remain a valid observation (T1 tissue times are not expected to vary much over 5 weeks). Results thus confirm elevated tumour [Na^+^] within the MDA-MB-231 preclinical breast cancer model.

### ^23^Na MRI improves the predictive potential of ADC for the classification of malignant lesions

**Fig. 2:**
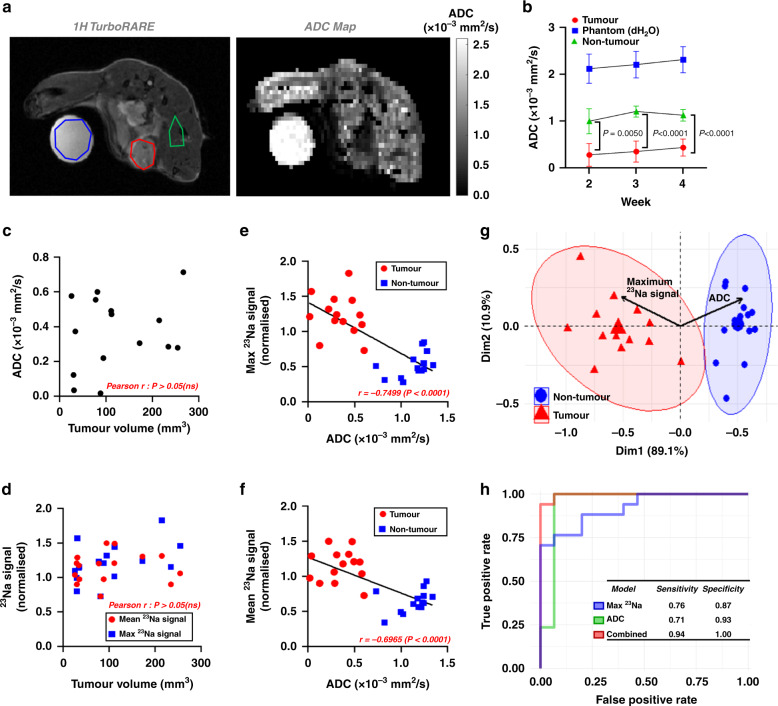
^23^Na MRI combined with and DWI improves tumour vs non-tumour classification accuracy. **a** Corresponding ^1^H TurboRARE images and reconstructed ADC maps (diffusion tensor imaging, *b* values of 100, 300 and 700 s/mm^2^) were obtained from mice bearing MDA-MB-231 xenograft tumours at 2, 3 and 4 weeks post implantation. Tumour (red), phantom (blue, 50 mM NaCl) and non-tumour (green) regions of interest (ROIs) are shown. **b** ADC in tumour, phantom and non-tumour regions at each imaging timepoint (week 2, *n* = 4; week 3, *n* = 5; week 4, *n* = 6. mean ± SEM). **c** Tumour volume does not correlate with tumour ADC (pooled ROIs across all timepoints, *n* = 15). **d** Neither mean nor maximum ^23^Na signal (^23^Na 2D cartesian acquisition) from tumour ROIs correlate with tumour volume (*n* = 16). Both maximum (**e**) and mean (**f**) ^23^Na signal negatively correlate with ADC across all regions (tumour, red; non-tumour, blue, all timepoints pooled, *n* = 30). **g** Principal component analysis biplot of maximum ^23^Na signal and ADC (data from **e**) with loading vectors for maximum ^23^Na signal and ADC with 95% concentration ellipses. Receiver-operating characteristics are shown (**h**) for linear discriminant models trained on maximum ^23^Na values alone, ADC alone, or combination (training data from e, test data from DTX control cohort (Fig. 5, *n* = 32 regions from all timepoints). Classification sensitivity and specificity are presented in the inset. *P*-values for significant differences between groups calculated using an unpaired, two-tailed Student’s *t* test and for correlation using a Pearson *r* test.

Low ADC correlates with high cellularity, malignancy and elevated tumour [Na^+^] [8–10, 38]; ^1^H DWI is used as an adjunct imaging modality for assessing malignant breast lesions in the clinic [7]. To determine whether tumour [Na^+^] is similarly an important biomarker of malignancy, we used machine learning approaches to compare the predictive power of tumour [Na^+^] vs ADC in classifying tumour (MDA-MB-231 xenografts) vs non-tumour regions. Across all timepoints, ADC was consistently significantly lower in tumour regions than in non-tumour regions (0.7-fold lower at week 3, Fig. 2a, b), as expected. Interestingly, tumour volume was not correlated with either ADC (Fig. 2c) or ^23^Na signal (Fig. 2d), indicating that both parameters were independent of tumour size. However, In line with clinical observations [8], ADC (all timepoints/regions pooled) was inversely correlated with both maximum ^23^Na signal (*r* = −0.750; Fig. 2e) and mean ^23^Na signal (*r* = −0.697; Fig. 2f).

To compare whether the mean or maximum ^23^Na signal performed better at classifying tumour vs non-tumour regions, we used data from Fig. 2e and f to train two single-variable LDA models and performed LOOCV. The maximum ^23^Na signal achieved a classification accuracy of 92.9%, whereas the mean ^23^Na signal achieved 89.3%. It was hypothesised that the reduced classification accuracy of mean ^23^Na signals was a consequence of tumour heterogeneity and/or partial volume effects due to slice thickness used. Notably, current technology levels and ^23^Na-MR signal insensitivity necessitates the use of low-resolution imaging and thick imaging slices to compensate and improve SNR. The maximum ^23^Na signal was therefore carried forward for comparisons with ADC. Principal component analysis of maximum ^23^Na signal and ADC reported clear separation of tumour and non-tumour regions (95% concentration ellipses, Fig. 2g). The distinct opposing direction and similar size of the associated loading vectors (Fig. 2g, black arrows) demonstrate that maximum ^23^Na signal and ADC are inversely correlated yet hold almost equivalent dominance along principal component 1, suggesting that these variables are equally important for explaining variance between the tissue groups.

To test the comparative predictive capacity of maximum ^23^Na signal vs ADC, we trained LDA models (data from Fig. 2e) and classified novel data taken from an independent experimental cohort (docetaxel vehicle group, Fig. 4). LDA models trained on maximum ^23^Na signal alone or ADC alone achieved accuracies of 81.2%, while the model trained on a combination of both parameters achieved an accuracy of 96.9%. The maximum ^23^Na signal or ADC single parameter models achieved sensitivities of 0.76 and 0.71 and specificities of 0.87 and 0.93, respectively, whereas the combined parameter model achieved a sensitivity of 0.94 and a specificity of 1.0. Moreover, the area under the ROC curve (AUC) for each of the models was 0.92 for ^23^Na, 0.95 for ADC, and 1 for the two parameters combined (Fig. 2h; confusion matrices presented in Supplementary Fig. 2). Together, these data indicate that the model that incorporates both parameters is optimal, with greater accuracy, specificity, and sensitivity for determining malignant lesions from non-tumour regions.

### Elevated tumour [Na^+^] is due to increased [Na^+^]_i_

**Fig. 3:**
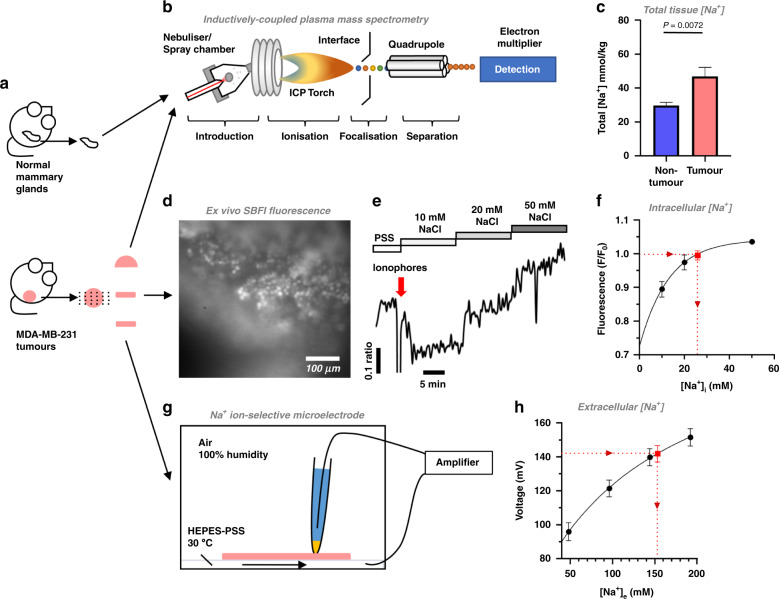
[Na^+^]_i_ is elevated in live xenograft tumour slices. **a** Following week-4 imaging, tumours were acutely isolated, divided and prepared for inductively coupled plasma mass spectrometry (ICP-MS, **b**) analysis or ex vivo measurements of [Na^+^]_i_ and [Na^+^]_e_. Healthy mammary glands (HMGs) from naive female mice were included in ICP-MS analysis. **c** Total tissue [Na^+^] in MDA-MB-231 xenograft tumours (*n* = 10) and HMGs (*n* = 11). **d** MDA-MB-231 xenograft tumour slices (200 µm) were loaded with SBFI-AM (10 µM) and immobilised for imaging. Baseline SBFI fluorescence was recorded during perfusion with HEPES-buffered physiological saline solution (PSS) containing 144 mM NaCl. **e** To calibrate [Na^+^]_i_ for each cell in situ, perfusion was switched to 10 mM NaCl, followed by application of the ionophores (red arrow) gramicidin (3 µM), monensin (10 µM) and ouabain (1 mM). Tumour slices were sequentially perfused with 20 mM and 50 mM NaCl. The final three frames acquired during each phase were used to determine the average fluorescence for each calibration step. **f** [Na^+^]_i_ during the baseline PSS phase (red point) was calibrated (*n* = 3 slices from two separate animals, 11–16 cells per experiment, F/F_0_) using non-linear regression (one-phase association). **g** Concurrently, Na^+^ ion-selective microelectrodes (ISMEs) calibrated with PSS of variable [Na^+^] (48 mM, 96 mM, 144 mM, 192 mM, no replacement ion) were used to measure [Na^+^]_e_ (red point) in MDA-MB-231 xenograft tumour slices (500 µm, *n* = 6 slices from six separate animals, 12 recordings per slice; **h**). Recordings were interpolated using non-linear regression (Padé (1,1) approximant). Data represent group mean ± SEM.

In agreement with ^23^Na MRI, inductively coupled plasma mass spectrometry (ICP-MS) identified significantly higher total tissue [Na^+^] in MDA-MB-231 xenograft tumour samples (46.9 ± 5.4 mM) compared with healthy mammary glands from naive mice (29.7 ± 1.9 mM, Fig. 3a − c). Identifying the compartmentalisation of this Na^+^ accumulation is critically important to determine its impact on tumour pathophysiology. Because low ADC in tumours indicates a larger intracellular volume fraction (IVF) compared with non-tumour regions, elevated tumour [Na^+^] is unlikely to reflect an increased extracellular volume fraction (EVF). We hypothesised that elevated tumour [Na^+^] instead reflects an increased [Na^+^]_i_. To test this, we used complementary ex vivo approaches to measure [Na^+^]_i_ and [Na^+^]_e_ within acutely isolated live MDA-MB-231 tumour slices.

The Na^+^ selective dye SBFI-AM (10 µM) and in situ calibration (dynamic range comparable to that previously described [33] Fig. 3d − f) revealed that resting [Na^+^]_i_ in tumour slices was 25.9 ± 1.0 mM (*n* = 3 slices, 11 − 16 cells per slice, two separate animals; Fig. 3f). This is higher than that expected in healthy mammalian cells (5 − 15 mM) [39] and yet is in line with previous in vitro fluorescence studies in cancer cell lines [20, 21, 40]. In contrast, Na^+^-selective microelectrodes [41] (Fig. 3g) showed that tumour slice [Na^+^]_e_ was 157.8 ± 1.0 mM (*n* = 6 slices, 12 recordings per slice, six separate animals; Fig. 3h). This is within the expected serum [Na^+^] range for female BALBc mice [42]. These results indicate that elevated tumour [Na^+^] is due to increased [Na^+^]_i_ while [Na^+^]_e_ is unchanged.

### Detection of docetaxel treatment response by ^23^Na MRI in MDA-MB-231 xenografts

Two prospective clinical studies with small-cohort sizes (*n* = 15 [12] and *n* = 5 [16] patients) found that elevated [Na^+^] in malignant breast lesions was reduced in patients responding to neoadjuvant chemotherapy. We sought to determine whether chemotherapy response can be detected using ^23^Na MRI in tumour-bearing mice using a randomised, controlled, interventional study approach.

**Fig. 4:**
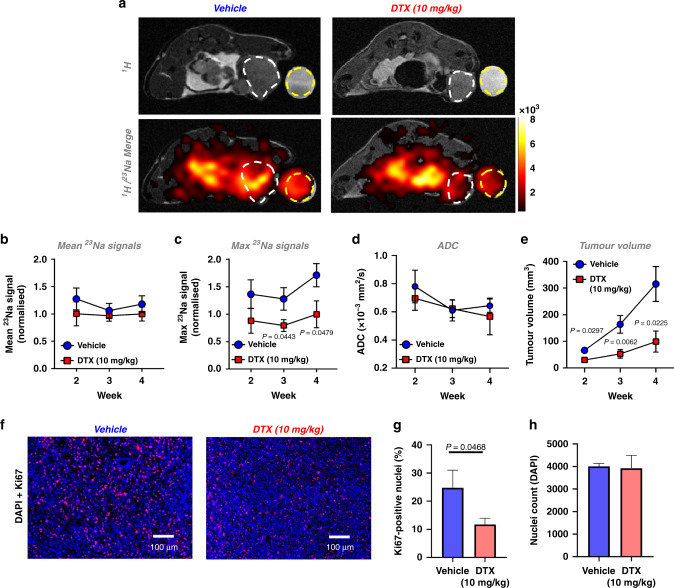
Docetaxel decreases the maximum ^23^Na signal detected within MDA-MB-231 xenograft tumours. Following orthotopic MDA-MB-231 cell implantation, *Rag2*^*−*/−^ *Il2rg*^*−*/−^ mice were randomised to receive either docetaxel (10 mg/kg) or vehicle i.p. once weekly. **a** Representative ^1^H TurboRARE and ^1^H/^23^Na 2D cartesian (merge) images for docetaxel and vehicle-treated mice at week 4 post implant; regions of interest (ROIs) for tumour (white dash) and NaCl phantom (50 mM, yellow dash) are annotated. Mean (**b**) and maximum (**c**) ^23^Na signals measured from tumour ROI at weeks 2, 3 and 4 post xenograft implantation (normalised to phantom to provide a concentration measurement. Vehicle, week 2, *n* = 7; week 3, *n* = 6; week 4, *n* = 7. Docetaxel, week 2, *n* = 9; week 3, *n* = 8; week 4, *n* = 7). **d** Effect of docetaxel on tumour ADC (Vehicle, week 2, *n* = 5; week 3, *n* = 6; week 4, *n* = 7. Docetaxel, week 2, *n* = 6; week 3, *n* = 8; week 4, *n* = 7). **e** Effect of docetaxel treatment on tumour growth rate (vehicle, *n* = 6 for all timepoints. Docetaxel, week 2, *n* = 8; week 3, *n* = 8; week 4, *n* = 5. Volume measured by multislice (1 mm) ^1^H TurboRARE imaging. **f** Representative images of Ki67/DAPI stained tissue tumour sections. **g** Ki67-positive nuclei (vehicle, *n* = 5; docetaxel, *n* = 7; 2–10 regions each section). **h** Nuclei counts (vehicle, *n* = 5; docetaxel, *n* = 6; 1–10 regions each section). Data represent group mean ± SEM. *P*-values for significant differences between groups calculated using an unpaired, two-tailed Student’s *t* test (**b**–**e**) or an unpaired, two-tailed nested *t* test (**g**, **h**).

Mice received docetaxel (10 mg/kg i.p.) or vehicle once weekly from week 1 post MDA-MB-231 xenograft implantation. Docetaxel treatment significantly inhibited tumour growth; at week 3, tumour volume within the treatment group was 32.2% of that within the vehicle group (Fig. 4e). Docetaxel treatment also significantly reduced Ki67-positive nuclei within tumour sections by 52.7% (Fig. 4f, g) despite no change in nuclei count (Fig. 4h). Moreover, docetaxel treatment conferred a significantly lower maximum ^23^Na signal within tumour regions compared with control mice (37.8% lower at week 3, Fig. 4a, c), suggesting that localised changes in tumour [Na^+^] reflect clinical response. However, no difference was observed between docetaxel and control groups for either mean tumour ^23^Na signal (Fig. 4b) or isolated tissue sample [Na^+^] (ICP-MS, Supplementary Fig. 3). The difference between the maximum and mean ^23^Na signals potentially reflects important intratumoural heterogeneity requiring improved MRI resolution to probe further.

Previous studies indicate that low ADC correlates with elevated Ki67 expression [43]. We performed DWI to assess whether treatment response was reflected in ADC. In agreement with the tumour section nuclei counts, there was no difference in ADC between the control and docetaxel groups (Fig. 4d). These data indicate that the docetaxel-induced change in tumour [Na^+^] was not due to altered cellularity, suggesting that tumour [Na^+^] might provide improved treatment monitoring over ADC.

### Effect of cariporide and eslicarbazepine acetate on tumour [Na^+^]

Many Na^+^ channels and transporters that utilise the inward electrochemical Na^+^ gradient are implicated in tumour progression [17]. Thus, elevated tumour [Na^+^]_i_ could reflect increased Na^+^ transporter activity. We, therefore, investigated whether inhibition of either NHE1 (cariporide) or VGSCs (eslicarbazepine acetate, ESL) reduces elevated [Na^+^] within MDA-MB-231 xenograft tumours. ESL and its major active metabolite (S)-licarbazepine [44] inhibit inward Na^+^ current in MDA-MB-231 cells [45], and was selected due to its preferred use in the clinic compared to older VGSC-inhibiting antiepileptic medications such as phenytoin [46]. Moreover, cariporide was administered at a dose that previously exhibited efficacy in in vivo models of cancer [26, 27].

**Fig. 5:**
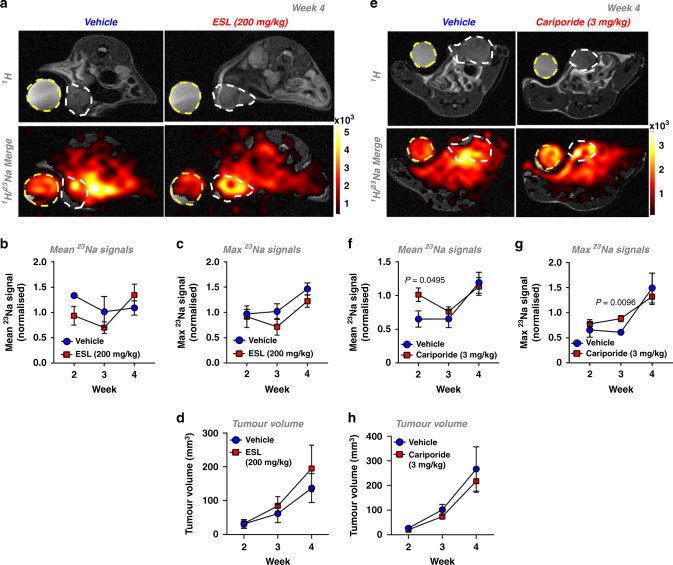
Cariporide and eslicarbazepine acetate have no effect on tumour [Na^+^] in MDA-MB-231 xenografts. *Rag2*^*−*/−^ *Il2rg*^*−*/−^ mice bearing MDA-MB-231 tumours were randomised to receive either cariporide (3 mg/kg) vs vehicle i.p. once daily or eslicarbazepine acetate (ESL, 200 mg/kg) vs vehicle p.o. once daily commencing 1 week post xenograft implantation. Representative ^1^H TurboRARE and ^1^H/^23^Na 2D cartesian (merge) images for (**a**) ESL vs vehicle (**e**) cariporide vs vehicle at week 4 post implant; regions of interest (ROIs) for tumour (white dash) and NaCl phantom (50 mM, yellow dash) are annotated. Mean (**b**, **f**) and maximum (**c**, **g**) ^23^Na signals from tumour ROIs (normalised to phantom to provide a concentration measurement) were measured at weeks 2, 3 and 4 post xenograft implantation (cariporide group: *n* = 5 per group per timepoint. ESL vehicle group: week 2, *n* = 3; week 3, *n* = 3; week 4, *n* = 4. ESL treatment group: week 2, *n* = 4; week 3, *n* = 4; week 4, *n* = 5). The tumour growth rate was measured by multislice ^1^H TurboRARE imaging (**d**, ESL. Vehicle, *n* = 4; treatment group, *n* = 5. **h** cariporide. Vehicle week 4, *n* = 4; all other data points *n* = 5). Data represent group mean ± SEM. *P*-values for significant differences between groups calculated using an unpaired, two-tailed Student’s *t* test.

ESL treatment (200 mg/kg daily p.o. from day 7 post implant) had no effect on the mean (Fig. 5a, b) or maximum (Fig. 5a, c) tumour ^23^Na signal compared with untreated control mice. Similarly, ESL had no effect on tumour volume (Fig. 5d). Cariporide (3 mg/kg daily i.p. from day 7 post implant) also did not reduce mean or maximum tumour ^23^Na signal compared with untreated control mice; in fact, there was a significant increase in mean ^23^Na signal at week 2 and maximum ^23^Na signal at week 3 (0.55- and 0.44-fold, respectively; Fig. 5e − g). Nevertheless, cariporide had no effect on tumour growth, in agreement with previous findings (Fig. 5h) [47]. There was also no effect of ESL (Supplementary Fig. 4a, b) or cariporide (Supplementary Fig. 4c, d) on VGSC gating or current density measured in ex vivo tumour slices. This was despite achieving plasma [licarbazepine] (1 hour post dosing: plasma, 6.5 ± 0.9 ng/µl; tumour, 4.5 ± 0.8 ng/mg, *n* = 3 tumours) comparable to that showing clinical antiepileptic efficacy [48]. Similarly, docetaxel had no effect on VGSC gating or current density in treated ex vivo tumour slices compared to controls (Supplementary Fig. 4e, f), suggesting that the docetaxel-induced change in tumour [Na^+^] is not mediated via an effect on VGSCs. Taken together, these data indicate that ESL and cariporide at the current doses elicit no detectable decrease in tumour [Na^+^], and suggest that targeting multiple Na^+^ transporters may be required to alter elevated tumour [Na^+^] in vivo.

### Tumour [Na^+^] is elevated across multiple murine models of breast cancer

**Fig. 6:**
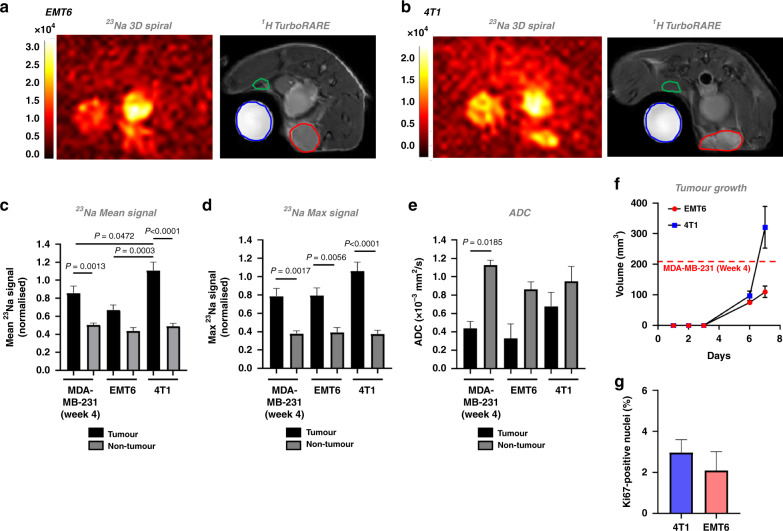
Elevated tumour [Na^+^] is a common feature of preclinical models of breast cancer. Representative ^23^Na 3D gradient-echo spiral out and ^1^H TurboRARE images are shown for orthotopic (**a**) EMT6 or (**b**) 4T1 allograft tumours, imaged at day 7 post implantation (tumour region of interest (ROI), red; non-tumour ROI, blue). Mean ^23^Na signal (**c**), and maximum ^23^Na signal (**d**) within tumour and non-tumour ROIs were normalised to NaCl phantom (50 mM) to provide a concentration measurement; data from MDA-MB-231 tumours at week 4 post implantation included for statistical comparison. ADC (**e**) was assessed by DWI. **f** Tumour volume (4T1 and EMT6) was calculated using the modified ellipsoidal formula volume = 1/2(length × width^2^); dotted line denotes the average size of MDA-MB-231 tumours at 4 weeks post implantation (see Supplemental Fig. 1a). **g** Ki67-positive nuclei (%) for 4T1 and EMT6 tumour sections (both groups *n* = 4, 3–12 regions each section). Data represent group mean ± SEM (4T1, EMT6, *n* = 5; MDA-MB-231, *n* = 6). *P*-values for significant differences between groups calculated using a one-way ANOVA with post hoc Tukey test for multiple comparisons (**c**, **d**), a Kruskal–Wallis test with post hoc Dunn’s test for multiple comparisons (**e**) or a nested *t* test (**g**).

To confirm the clinical relevance of these findings, we next assessed whether elevated tumour [Na^+^] is a common feature across other orthotopic murine models of breast cancer. We selected the EMT6 (Fig. 6a) and 4T1 (Fig. 6b) murine mammary carcinoma cell lines, which form solid tumours in immunocompetent BALB/c mice. These cell lines give rise to luminal A tumours that are weakly ER-positive, claudin-low (EMT6) and ER-negative and non-claudin low (4T1). Moreover, these models exhibit significant immunogenicity and leucocytic infiltration, with EMT6-derived tumours exhibiting higher GR1 + granulocyte and CD3 + T-cell infiltration [49].

EMT6 and 4T1 tumours grew rapidly after implantation (Fig. 6f), reaching a similar size at 1 week post implant to MDA-MB-231 cells at 4 weeks post implant; no difference was observed in Ki67 density between the two models (Fig. 6g). At 1 week post implant, ^23^Na MRI revealed elevated [Na^+^] in both EMT6 (Fig. 6a) and 4T1 (Fig. 6b) tumours. In 4T1 tumours, mean ^23^Na signal was significantly elevated compared with non-tumour regions (0.5-fold increase, Fig. 6c). Similarly, maximum ^23^Na signal was significantly elevated (onefold increase, EMT6; 1.8-fold increase, 4T1; Fig. 6d) within tumour regions compared with non-tumour regions. Interestingly, 4T1 tumours exhibited significantly higher mean ^23^Na signal compared with EMT6 or MDA-MB-231 tumours (0.7-fold and 0.2-fold increases, respectively; Fig. 6c). These results suggest that although elevated tumour [Na^+^] is consistent across multiple models of breast cancer, the absolute tumour [Na^+^] is variable, hinting at important underlying biological differences in tumour Na^+^ handling. However, in contrast to the MDA-MB-231 model, no significant difference was observed in ADC between tumour and non-tumour regions in EMT6 and 4T1 models (Fig. 6e). Similar to [Na^+^], these data suggest that tumour ADC is variable across these models. Importantly, however, given that elevated tumour [Na^+^] was identified by ^23^Na MRI in the 4T1 model but no difference was seen in ADC, ^23^Na MRI might in certain cases be better positioned than DWI to discern malignancy.

## Discussion

This study is the first to demonstrate that tumour [Na^+^] is elevated relative to non-tumour regions in the orthotopic MDA-MB-231 xenograft and 4T1 and EMT6 allograft models of breast cancer. Moreover, while supervised classification algorithms trained on ^23^Na MRI data achieved similar accuracy to those trained on DWI data (ADC) in distinguishing tumour vs non-tumour regions, a model trained on both parameters combined achieved superior classification accuracy, suggesting that ^23^Na MRI holds promise as a novel diagnostic readout. Using a randomised, controlled study approach, we show that docetaxel, a clinically used antineoplastic drug that disrupts microtubule formation, reduces maximum tumour [Na^+^] while slowing tumour growth rate but has no effect on ADC. These findings suggest that ^23^Na MRI also has utility as a readout of response to chemotherapy, and may be better suited for this purpose than DWI. These findings broadly confirm for the first time in a randomised, controlled, interventional study design, the chemotherapy response effect reported in previous small-cohort clinical observational studies [12, 13]. We show in intact breast tumour slices that elevated tumour [Na^+^] reflects increases in [Na^+^]_i_, while [Na^+^]_e_ is unaltered. This aberrant intracellular Na^+^ handling sheds light on a previously underappreciated characteristic of malignant breast lesions and has considerable implications for pathophysiological processes linking ion homoeostasis to tumour cell function.

The finding that tumour [Na^+^] is elevated within mouse models of breast cancer is consistent with previous clinical breast cancer studies [8, 12, 13]. Ouwerkerk et al. reported that mean total tissue [Na^+^] was ~53 mM (*n* = 19 patients) in malignant lesions and ~26 mM in benign lesions (*n* = 3 patients) [13]. Similarly, Zaric et al. reported a mean total tissue [Na^+^] of ~69 mM in malignant lesions compared with ~35 mM in normal glandular tissue (*n* = 17 patients) [8]. These values are broadly in agreement with our ICP-MS and ^23^Na MRI data. However, our finding that maximum ^23^Na signal improves distinction of tumour vs non-tumour regions compared to mean ^23^Na signal potentially reflects considerable heterogeneity of [Na^+^] within tumours. Indeed, despite the limited spatial resolution, we observed clear variability in ^23^Na signal across the tumour regions (Figs. 1, 4, and 6). This heterogeneity could also explain why docetaxel treatment conferred a reduced maximum ^23^Na signal within tumours despite no difference in mean ^23^Na signal. This has broader implications for prognosis, since intratumoural heterogeneity is considered a key contributor to resistance to chemotherapy [50], and suggests a potential link between local changes in tumour [Na^+^] and cancer cell function that could be discerned using ^23^Na MRI. The realisation of improved spatial specificity for ^23^Na MRI would enable incorporation of tumour [Na^+^] heterogeneity within more complex analyses. Indeed, a recent study coregistering tumour section histology with ^1^H parameters (including DCE MRI and DWI) found that DCE MRI could distinguish distinct tumour subregions [51]. Inclusion of (or replacement with) non-invasive ^23^Na readouts would offer an important additional dimension to such functional imaging methods. We note that future work should quantify ^23^Na T_1_ relaxation parameters to better describe absolute millimolar tissue concentrations from ^23^Na MRI data. However, current technology limits and the insensitivity of ^23^Na MRI necessitates the use of short TR times to increase averages to gain SNR - especially when multiparametric measures (e.g. diffusion, ^23^Na and structural imaging) are required from the same animal model. Future work could also explore use of Tm^3+^ based complexes/shift reagents with chemical shift-based imaging to separate blood pool, intra and extracellular compartments of the Na^+^ signal [52].

This study establishes that elevated tumour [Na^+^] coregisters with a low ADC within preclinical models of breast cancer, and that these parameters are inversely correlated across non-cancerous and malignant lesions, recapitulating previous clinical observations [8]. These findings suggest a functional link between high cellularity and Na^+^ accumulation in tumours, supporting the notion that elevated tumour [Na^+^] is likely due to changes within the intracellular compartment. Interestingly, principal component analysis revealed that ADC and maximum ^23^Na signal held almost equivalent importance when explaining variance across all regions (tumour and non-tumour). LDA models trained on either ^23^Na signal or ADC were comparable in their ability to correctly classify regions. However, a model trained on both variables achieved superior classification accuracy. One can therefore hypothesise that their predictive resolutions do not completely overlap (see Supplementary Fig. 2) and are complementary.

While recent recommendations have been published [53], there is as yet no clearly established consensus on optimal DWI methodology or a threshold ADC value with which to denote malignancy [54]. DWI sensitivity and ADC quantification are influenced by differences in methodology (i.e., choice of *b* values, magnet strength) and tumour heterogeneity [55, 56], thereby preventing the establishment of a generalised threshold ADC value. Moreover, a negative correlation between ADC and high cellularity is not universally apparent in all cancer subtypes [38]. Taken together with the improved statistical discrimination of 4T1 and EMT6 tumours by ^23^Na MRI vs DWI, our classification results suggest that combining DWI with ^23^Na MRI could improve specificity and predictive accuracy in the clinic, enabling superior non-invasive diagnostic and treatment monitoring beyond DWI alone.

This study is the first to report an effect of docetaxel on tumour [Na^+^] in the preclinical setting. Two small, prospective clinical studies in patients with breast cancer previously measured significant reductions (27%, *n* = 15 patients [12]; 21%, *n* = 5 patients [16]) in total tissue [Na^+^] using ^23^Na MRI following response to neoadjuvant chemotherapy. In this study, docetaxel treatment conferred slower tumour growth and decreased maximum tumour ^23^Na signals (37.8% lower than the vehicle group at week 3). However, we do not currently know the mechanism underlying this, nor whether the decrease in tumour [Na^+^] contributes to, or is a consequence of, treatment response. Nonetheless, treatment with docetaxel did not decrease ADC, suggesting a mechanism other than altered cellularity; for example, decreased tumour vascularity (docetaxel is antiangiogenic). These data suggest that, in certain circumstances, ^23^Na MRI may be better positioned to discern treatment response than DWI.

Beyond breast cancer, contrasting observations have been made in fibrosarcoma and gliosarcoma models where cyclophosphamide or carmustine treatment, respectively [57, 58] led to a correlated increase in both ^23^Na signal and ADC. The authors concluded that ^23^Na signal and ADC both report the same physiological event, namely decreased cellularity and increased EVF. This is in contrast to the present study, where tumour ADC was unchanged yet maximum ^23^Na signal decreased following docetaxel treatment. These conflicting findings suggest that tumour [Na^+^] responses differ depending on cancer type and choice of chemotherapeutic intervention.

This study provides direct evidence from intact, live MDA-MB-231 tumour slices that elevated tumour [Na^+^] arises due to an intracellular accumulation of Na^+^; [Na^+^]_i_ was 25.9 mM within the tumour slices (expected upper physiological bound for [Na^+^]_i_ is ~15 mM [39]). This is in agreement with our DWI results, which suggest that EVF is decreased in tumours vs non-tumour regions, and that elevated tumour [Na^+^] must therefore reflect an increase in either [Na^+^]_i_ or [Na^+^]_e_. Moreover, tumour slice [Na^+^]_e_ was within the expected physiological range for female BALB/c mouse serum (~157 − 160 mM [42]), indicating that elevated tumour [Na^+^] is not due to changes in [Na^+^]_e_. Interestingly, our observed [Na^+^]_i_ is similar to that recently extrapolated (~30 mM) from complementary ^23^Na and ^1^H DCE MRI approaches in patients with triple-negative breast cancer [59]. Furthermore, although at the limit of statistical distinction, intracellular-weighted ^23^Na imaging determined that [Na^+^]_i_ is elevated in prostate tumours compared with adjacent healthy tissue [60]. Given the small size of MDA-MB-231 xenograft tumours, similar [Na^+^]_i_ measurements could be enabled by improvements to ^23^Na contrast to noise ratio that are subsequently sacrificed to increase spatial resolution [61].

Using a simplified model, the relative EVF to IVF of xenograft tumours can be estimated from values obtained from the present study. If total tissue [Na^+^] = (1 − EVF) × [Na^+^]_i_ + EVF × [Na^+^]_e_, where EVF = 1 − IVF [13], EVF and IVF can be resolved for given values of [Na^+^]_i_, [Na^+^]_e_ and total tissue [Na^+^]. For our tumour slice [Na^+^]_i_ and [Na^+^]_e_ values (25.9 and 157.8 mM, respectively) and the observed total tumour [Na^+^] (46.9 mM), we expect an EVF of 0.159 and IVF of 0.841. Keeping these EVF and IVF values constant and rearranging the above equation for healthy mammary gland [Na^+^]_i_ (total tissue [Na^+^] = 29.7 mM) with an assumed [Na^+^]_e_ of 158 mM [42] returns a value of 5.4 mM; this is within the expected physiological range [39]. However, this simplified model does not take into account changes to the EVF:IVF ratio in tumours relative to healthy tissue, nor the blood or ductal compartments. Using shift reagents, one can quantify the contribution of these compartments with magnetic resonance spectroscopic imaging [62]; however, this would be invasive and therefore of limited clinical use.

An elevated [Na^+^]_i_ has important implications for cancer cell function, as it would impact upon processes governed by Na^+^-linked channels and transporters [17]. For example, Na^+^/K^+^ ATPase is the predominant consumer of intracellular ATP [63], and its activity increases across the [Na^+^]_i_ physiological range (5 − 15 mM) [64], suggesting full activation in tumours exhibiting a [Na^+^]_i_ of ~26 mM. This has important implications for aberrant cancer metabolism [17], Similarly, mitochondrial Na^+^/Li^+^/Ca^2+^ exchanger activation by elevated [Na^+^]_i_ (K_m_ ~ 12 mM) [65] could alter mitochondrial function and bioenergetics. Processes altered by elevated [Na^+^]_i_ could therefore represent novel therapeutic loci.

On the other hand, the elevated [Na^+^]_i_ could reflect increased Na^+^ channel or transporter activity [17]. For example, cancer cell pH and migration are regulated by the activity of NHE1 [25], and VGSCs aberrantly expressed in MDA-MB-231 cells pass a persistent inward Na^+^ current and regulate metastasis [23, 29–31]. Pharmacological data suggest that functional VGSCs may also be present in 4T1 cells [66]; however, their expression status in EMT6 cells is unknown. Plasma membrane Na^+^ channels and transporters are attractive pharmacological targets as they are easily accessible to small-molecule inhibitors and are already targeted by clinically approved drugs. However, in contrast to docetaxel, selective inhibition of either NHE1 or VGSCs (cariporide or ESL, respectively) did not reduce elevated tumour [Na^+^] in the present study at clinically relevant doses. On the other hand, we saw no effect of docetaxel treatment on VGSC gating properties or peak current density in ex vivo tumour slices, despite a decrease in maximum tumour ^23^Na signal (evidence suggests taxanes can modulate VGSC activity in MDA-MB-231 cells [67]). Although we cannot rule out subtle, localised changes in [Na^+^] beyond the sensitivity of the present MRI approach, these results suggest that alternative Na^+^-dependent transporters are responsible for elevated tumour [Na^+^]_i_ [17].

In conclusion, this study highlights ^23^Na MRI as a novel diagnostic biomarker for breast cancer diagnosis and readout of response to chemotherapy. In addition, we elevate the importance of altered [Na^+^]_i_ handling in breast tumour pathophysiology, and identify altered tumour [Na^+^] as a common characteristic of preclinical breast cancer models. The inclusion of non-invasive ^23^Na MRI in the patient care pathway may therefore represent an important clinical refinement in the fight against breast cancer. Supported by these results, the future development of non-invasive ^23^Na MRI with improved contrast to noise ratio [61] (sacrificed to increase spatial resolution) will further empower its use for diagnostics and exploring tumour heterogeneity and function.

## Supplementary information

Supplementary Methods and Figure Legends

Supplemental Figure 1

Supplemental Figure 2

Supplemental Figure 3

Supplemental Figure 4

Reproducibility checklist

ARRIVE Guidelines completed checklist

## Data Availability

All analysed and derivative raw data are available on request.
