# Supplementary material for: Sodium accumulation in breast cancer predicts malignancy and treatment response
Source: Br J Cancer. 2022 Apr 25;127(2):337–49. doi: 10.1038/s41416-022-01802-w (PMC9296657; doi:10.1038/s41416-022-01802-w)
Supplement: Supplementary file 1 — Supplementary Methods and Figure Legends [file 41416_2022_1802_MOESM1_ESM.docx]

**Supplementary Methods**

## ***Cell culture***

MDA-MB-231 cells, a gift from M. Djamgoz (Imperial College London), were cultured in Dulbecco’s modified Eagle’s medium (DMEM 219690-35, Thermo Fisher Scientific) supplemented with 5% foetal bovine serum (FBS) and 4 mM L-glutamine, and were authenticated by short tandem repeat analysis. EMT6 cells (European Collection of Authenticated Cell Cultures) were cultured in Minimum Essential Medium Eagle’s containing Earle’s Balanced Salt Solution (EMEM M2279, Sigma Aldrich, UK) supplemented with 2 mM glutamine,1% Non Essential Amino Acids (NEAA) and 10% FBS. 4T1 cells were a gift from Dr. Mihaela Lorger (University of Leeds UK, sourced from ATCC), and were cultured in EMEM supplemented with 2 mM L-glutamine, HyClone™ Vitamin Solution (Cytiva, 1x final working concentration), 1% NEAA, 1 mM sodium pyruvate and 10% FBS.

## ***Ion Selective Microelectrodes***

To produce Na^+^ ion-selective microelectrodes (ISMEs), non-filamented borosilicate micropipettes were pulled to a resistance of 5-10 MΩ (P-97 micropipette puller, Sutter Instruments, Novato, CA, USA) and coated with a hydrophobic layer of N,N-dimethyltrimethylsilylamine (Sigma Aldrich, UK). Micropipettes were back-filled with HEPES-PSS containing 144 mM NaCl, 5.4 mM KCl, 1 mM MgCl_2_, 2.5 mM CaCl_2_, 5 mM HEPES and 5.6 mM glucose, (adjusted to pH 7.2 with NaOH) and front-filled by suction with oil containing Na^+^ ionophores (sodium ionophore II – cocktail A Selectophore™, Sigma Aldrich, UK). An Axon 900B amplifier (Molecular Devices, Sunnyvale, CA) with a 200 GΩ impedance HS-2 headstage was used to measure voltage. Signals were low-pass filtered at 10 Hz and digitised by an NPI LHBF-48X amplifier/filter system (npi electronic GmbH, Bauhofring, Germany). Tissue slices (400 µm thickness) were held at the interface between perfused PSS and 100% humidified air at 30 °C during ISME recording.

##

## ***Ex vivo SBFI fluorescence imaging***

Slices were immobilised within a perfusion chamber (RC-26G, Harvard Bioscience) using a slice hold down (SHD-26GH/10, Harvard Bioscience), and were mounted on an imaging system comprised of a TE200 microscope fitted with a Plan Fluor ELWD 20x/0.45 Ph1 objective (Nikon Corporation, Tokyo, Japan) and a Rolera XR 12 Bit Fast 1394 CCD camera (QImaging, Surrey, British Columbia) controlled by SimplePCI software (Hamamatsu). Excitation light (340 and 380 nm, 50 ms exposure) was separated from emitted light using a 400 DCLP dichroic with a D510/80m filter.

***MRI***

^1^H and ^23^Na MR imaging was achieved using either i) a decoupled ^1^H quadrature volume resonator (Bruker, 300 MHz, 1 kW max, outer diameter 114 mm/inner diameter 72 mm) and bespoke, 3 cm ^23^Na surface coil; or ii) a dual-tuned linear ^1^H/^23^Na volume coil (inner diameter 35 mm, outer diameter 112 mm, coil length 50 mm, RAPID Biomedical GmbH, Rimpar).

Following ^1^H localiser scans (IntraGate based), all ^23^Na imaging to assess total tissue Na^+^ utilised either a 2D gradient echo Cartesian acquisition sequence (2D Cartesian, ^23^Na surface coil and ^1^H/^23^Na volume coil) or a 3D gradient echo spiral out sequence (3D spiral, ^1^H/^23^Na volume coil only) for readout.

2D Cartesian: TE, 1.512 ms; TR, 50 ms; ɑ, 90°; nominal resolution, 32x32 voxels; FOV, 40x40 mm; 400 averages; 10 minutes 40 seconds acquisition duration. Slice thickness was determined according to the diameter of the tumour (4 - 10 mm) to maximise in-plane signal.

3D spiral: TE, 1.533 ms; TR, 10 ms; ɑ, 90°; nominal resolution, 50x50x8 voxels; FOV, 30x30x30 mm; 100 averages; 10 minutes 40 seconds acquisition duration, 3D stack of spirals. The image volume was centred such that a single ‘slice’ covered the majority of the tumour (based on the localiser scans).

Complementary, slice geometry matched, high resolution (256*256) ^1^H images were taken (cartesian readout). A TurboRARE sequence was used with TR, 2500 ms; TE (effective), 33 ms; RARE factor 8; 2 averages; slice thickness 1 mm; with the number of slices determined by the ^23^Na-MRI image block (e.g. 8 slices if 2D Cartesian slice thickness was 8 mm). DWI was performed with a linear readout-segmented (8x) echo-planar spin echo imaging sequence and Stejskal-Tanner diffusion gradients applied either i) in three perpendicular directions (MDA-MB-231 xenografts, Figures 2, 4 and 5, b values 100, 300 and 700 s/mm^2^) or ii) one direction (EMT6, 4T1 allografts, Figure 6, 100 to 800 s/mm^2^ in increments of 100 s/mm^2^); b values were selected to maximise the contrast-to-noise ratio between tumour and non-tumour tissue as previously described [(1)](https://paperpile.com/c/ikl7or/SXLjA). Diffusion gradient duration was 4 ms; gradient separation was 10 ms. In all cases five A_0_ images were acquired (b=0); General sequence parameters were as follows: TR, 2000 ms; TE, 34.6 ms; averages 1; slice thickness, 2 mm (covering the centre of the tumour); Bandwidth, 300 kHz.

## ***Inductively-coupled plasma mass spectrometry (ICP-MS)***

Before digestion, tumour and healthy mammary gland samples were freeze dried overnight (PowerDry LL1500) and both wet and dry weights recorded. The dried sample was digested with nitric acid and hydrogen peroxide (4:1, trace metal grade) within a PTFE digestion vessel using an ETHOS UP microwave digestion system (Milstone, Bergamo, Italy) and the digestion method "SK-CL-002-Animal Tissue". Digestate was diluted to volume using purified H_2_O. Trace metal contamination from glassware was eliminated using a traceClean acid-steam cleaning system (Milestone). Na^+^ content was quantified using certified reference standards (multi-element environmental calibration standard, Agilent, 5183-4688) and a 10 ppb internal standard solution prepared from a certified reference solution (Agilent, 5188-6526). To determine [Na^+^], the weight (g) of Na^+^ per kg of fresh weight tumour was calculated from the parts per thousand Na^+^ concentration of the dry sample multiplied by the dry sample weight to wet sample weight ratio. This value was then divided by the MW of Na^+^ to give [Na^+^] in mol/kg (reported as mmol/kg).

***LC-MS analysis of plasma and tumour [licarbazepine]***

Lysates were centrifuged (4800 rpm, 4ᵒC, 15 min) and protein was precipitated from the supernatant by mixing lysate with acetonitrile (1:20) containing internal standard (10,11-dihydrocarbamazepine, 10.5 ng/ml, Sigma Aldrich, UK), vortexing, and incubating at -20ᵒC for 30 min. Precipitated protein was removed (centrifuged at 10000 rcf for 7 min at 4ᵒC) and the supernatant analysed by LC-MS (injection volume: 2 µl). Using a 6-level calibration curve of licarbazepine (3.7–900 ng/ml, Tocris Bioscience), normalised data were quantified using a quadratic equation fit (R^2^ = 1.0). Limit of detection and limit of quantification were 0.64 and 1.91 ng/ml, respectively. The LC-MS system consisted of an Acquity I-Class LC (Waters, Elstree, UK) and a Waters BEH C18 column (50 x 2.1 mm, 1.7 µm) including a filter frit, maintained at 40ᵒC. Sample temperature was 10ᵒC). Mobile phase consisted of A) 10% (v/v) acetonitrile with 0.1% acetic acid, and B) 90% acetonitrile with 0.1% acetic acid. LC-MS gradient was 0% B 0 min, 40% B 3 min; 100% B 3.1 -5.5 min, 0% B 5.6–7 min. The LC was connected to a Synapt G2-Si (Waters, Elstree, UK) operating in positive electrospray ionisation sensitivity mode (capillary voltage, 0.5 kV; sampling cone voltage, 40 V; source and desolvation temperatures, 150ᵒ and 500ᵒC; cone gas, 10 L/h; desolvation gas, 1000 L/h; nebuliser, 6.5 bar). Precursor/fragment pairs in the multiple reaction monitoring (MRM) scans were 237.12/194.10 for LIC, 239.13/194.10 for DHC. Data were analysed using Skyline 20.2.0 [(2)](https://paperpile.com/c/ikl7or/nrGZl).

##

## ***Patch-clamp electrophysiology***

Plasma membrane Na^+^ currents in cells within acutely isolated tumour slices (200 µm, sliced with a vibratome as described above) were recorded using the patch clamp method in the whole cell configuration, as previously described [(3,4)](https://paperpile.com/c/ikl7or/yo2kC+yBVXz). Borosilicate glass patch pipettes were pulled (P-97 pipette puller, Sutter Instruments) and fire polished to achieve a resistance of 3–5 MΩ when filled with intracellular recording solution (containing 5 mM NaCl, 145 mM CsCl, 2 mM MgCl_2_, 1 mM CaCl_2_, 10 mM HEPES and 11 mM EGTA, (adjusted to pH 7.4 with CsOH) and bathed in HEPES-PSS. Recordings were performed using a Multiclamp 700B amplifier (Molecular Devices) at room temperature, compensating for series resistance by 40-60%. A Digidata interface (Molecular Devices) was used to digitize currents, which were low pass filtered at 10 kHz and sampled at 50 kHz. All recordings were analysed using pCLAMP 10.7 software (Molecular Devices).

Two voltage clamp protocols were used:

1. To determine the voltage-dependence of activation, cells were held at -120 mV for 250 ms and then depolarised to between -85 mV and +30 mV for 50 ms (5 mV steps).
2. To determine the voltage-dependence of steady-state inactivation, cells were held at -120 mV for 250 ms, and then sequentially held at a prepulse potential between -120 mV and +30 mV for 250 ms (5 mV steps) followed by a test pulse to -10 mV for 50 ms.

The voltage-dependence of activation and steady-state inactivation were determined as follows:

1. Voltage-dependence of activation: G = I/(V_m_ – V_rev_), where G is conductance, I is current, V_m_ is the membrane voltage and V_rev_ is the reversal potential for Na^+^ (+84.64 mV, derived from the Nernst equation). Conductance was normalised G/G_max_.

2. Voltage-dependence of steady-state inactivation: Current was normalised I/I_max_.

Both data sets were fitted to Boltzmann sigmoidal curves. The resulting V_1/2_ + k values (individual cells) were compared between groups using an unpaired, two-way Student’s t-test.

**References for supplementary methods**

1. [Bogner W, Gruber S, Pinker K, Grabner G, Stadlbauer A, Weber M, et al. Diffusion-weighted MR for differentiation of breast lesions at 3.0 T: how does selection of diffusion protocols affect diagnosis? Radiology. 2009 Nov;253(2):341–51.](http://paperpile.com/b/ikl7or/SXLjA)

2. [Adams KJ, Pratt B, Bose N, Dubois LG, St John-Williams L, Perrott KM, et al. Skyline for Small Molecules: A Unifying Software Package for Quantitative Metabolomics. J Proteome Res. 2020 Apr 3;19(4):1447–58.](http://paperpile.com/b/ikl7or/nrGZl)

3. [Nelson M, Yang M, Millican-Slater R, Brackenbury WJ. Nav1.5 regulates breast tumor growth and metastatic dissemination in vivo. Oncotarget. 2015 Oct 20;6(32):32914–29.](http://paperpile.com/b/ikl7or/yo2kC)

4. [Yang M, Kozminski DJ, Wold LA, Modak R, Calhoun JD, Isom LL, et al. Therapeutic potential for phenytoin: targeting Na(v)1.5 sodium channels to reduce migration and invasion in metastatic breast cancer. Breast Cancer Res Treat. 2012 Jul;134(2):603–15.](http://paperpile.com/b/ikl7or/yBVXz)

**Supplementary Figure Legends**

**Supplementary Figure 1**: **Validation of MDA-MB-231 xenograft model and ^23^Na MRI acquisition sequences.** (a) Growth rate of orthotopic MDA-MB-231 tumour xenografts; cells seeded at 5 x 10^5^ cells per mouse, 50:50 PBS:Matrigel (n=6, volume measured by multislice (1 mm) ^1^H TurboRARE imaging at Weeks 2, 3 and 4 post-implant). The tumour doubling time was 4.75 days. (b) Axial slice of six 4 mm diameter phantoms containing known NaCl concentrations (1% agarose, [NaCl] in mM annotated) and corresponding representative ^23^Na images using ^23^Na FLASH (c) and ^23^Na spiral (d) acquisition. (e) ^23^Na signal (normalised to that of 50 mM NaCl) was linear with [NaCl] for both the ^23^Na FLASH (blue) and ^23^Na spiral (red) acquisition (^23^Na FLASH: R^2^ = 0.7856; 0 mM and, 100 mM, n=3 pixels; 20–80 mM, n=4 pixels. ^23^Na spiral: R^2^ = 0.8565; 0 mM, n=8 pixels; 20–80 mM, n=4 pixels; 100 mM, n=6 pixels). Data represent group mean ± SEM.

**Supplementary Figure 2**: **Confusion matrices for sensitivity and specificity values presented in Figure 2h.** Linear discriminant analysis models were trained on maximum ^23^Na values alone, ADC values alone, or a combination of both parameters, using region of interest (ROI) data from tumour and non-tumour regions (data from Figure Ei, n=30 regions from all timepoints). These models were then used to classify regions from the independent docetaxel cohort vehicle group (data from Figure 5, n=32 regions from all timepoints).

**Supplementary Figure 3: Analysis of tumour tissue [Na^+^] by ICP-MS following treatment with docetaxel.** Total tumour [Na^+^] from acutely isolated (Week 4) MDA-MB-231 tumour xenograft samples treated with either docetaxel (10 mg/kg once weekly i.p. from day 7, n=3) or vehicle (n=4), as determined by inductively-coupled plasma mass spectrometry. Data represent mean ± SEM.

**Supplementary Figure 4.** **Eslicarbazepine acetate (ESL), cariporide and docetaxel have no effect on voltage gated Na^+^ channel current in acutely isolated MDA-MB-231 tumour slices.** Activation and steady-state inactivation (SSI) were determined for voltage-gated Na^+^ channels within intact MDA-MB-231 tumour slices from animals treated with either ESL vs vehicle (a, red squares, ESL 200 mg/kg p.o. once daily (SSI, n=17; activation n=20) beginning day 7 post implant; vehicle (SSI, n=14; activation, n=16) shown in black squares), cariporide vs vehicle (c, red squares, cariporide 3 mg/kg i.p. once daily (SSI and activation, n=11) beginning day 7 post implant; vehicle (SSI and activation, n=9) shown in black squares) or docetaxel vs vehicle (e, red squares, docetaxel 10 mg/kg i.p. once weekly (SSI, n=14; activation, n=15) beginning day 7 post implant; vehicle (SSI, n=23; activation, n=24) shown in black squares). Tumour slices (200 µm) were isolated following Week 4 MRI. To determine SSI, a holding potential of -120 mV was applied, followed sequentially by 250 ms conditioning voltage prepulses between -105 and +30 mV (5 mV steps) and 50 ms test pulses at -10 mV; the resulting normalized currents (I/I_max_) were plotted as a function of the prepulse voltage. To determine activation, a holding potential of -120 mV was applied, and currents elicited by 5 mV depolarising steps from -85 to +30 mV for 30 ms; normalized conductance (G/G_max_) was calculated from current and plotted as a function of voltage, Both activation and SSI curves fitted with Boltzmann functions. Peak current densities were quantified in slices from tumours treated with either (b) ESL (n=20) or vehicle (n=16) (d) cariporide (n=11) or vehicle (n=9), or (f) docetaxel (n=15) or vehicle (n=24). Data represent mean ± SEM.
