# Supplementary figures and images for: Sodium accumulation in breast cancer predicts malignancy and treatment response
Source: Br J Cancer. 2022 Apr 25;127(2):337–49. doi: 10.1038/s41416-022-01802-w (PMC9296657; doi:10.1038/s41416-022-01802-w)

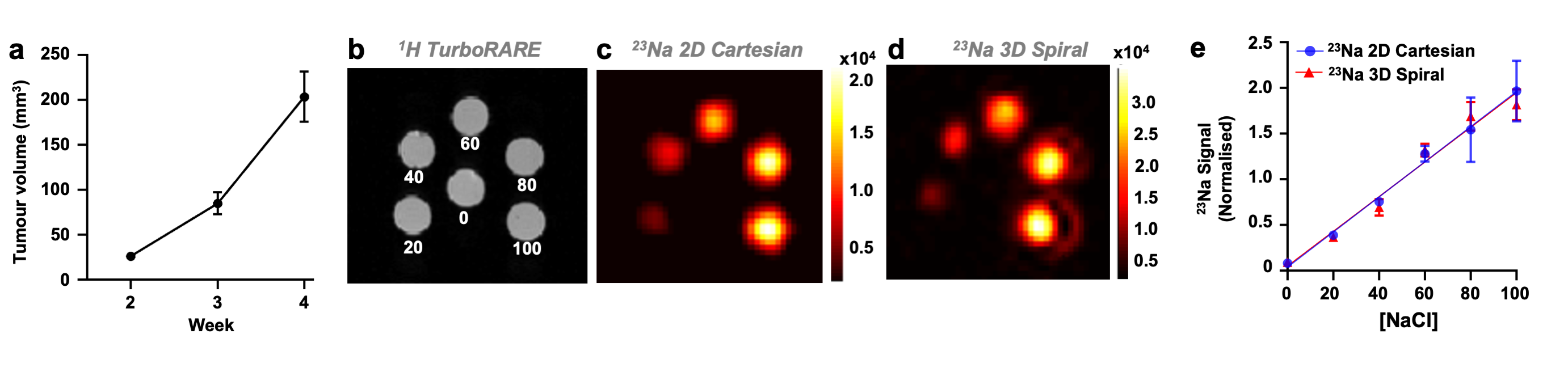

Supplement: Supplementary file 2 — Supplemental Figure 1 [file 41416_2022_1802_MOESM2_ESM.tif]

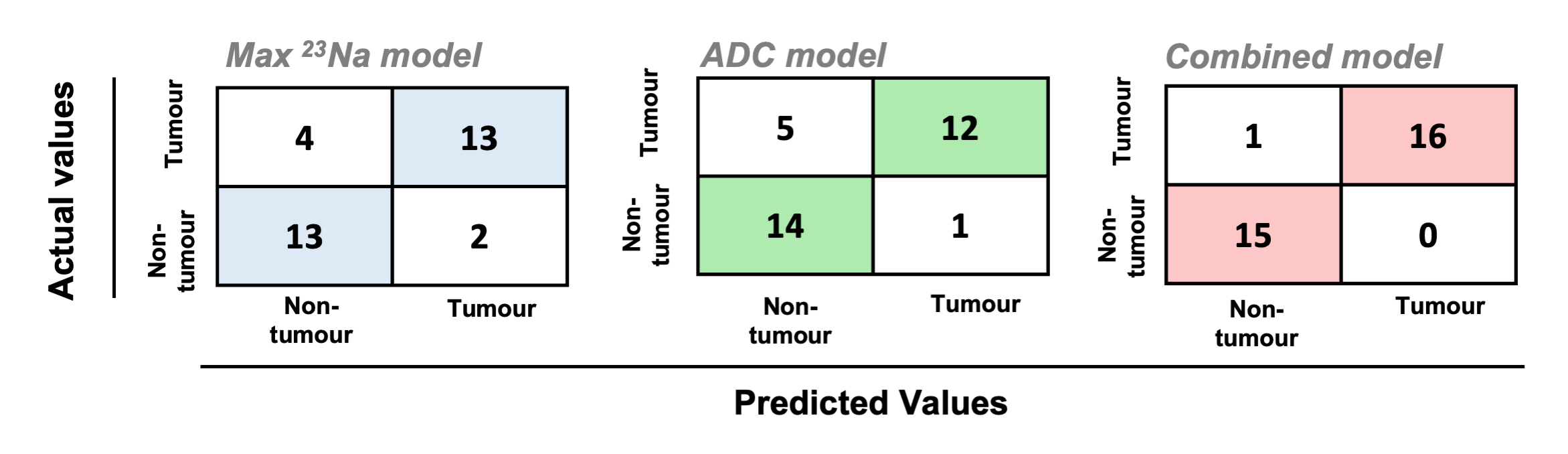

Supplement: Supplementary file 3 — Supplemental Figure 2 [file 41416_2022_1802_MOESM3_ESM.tif]

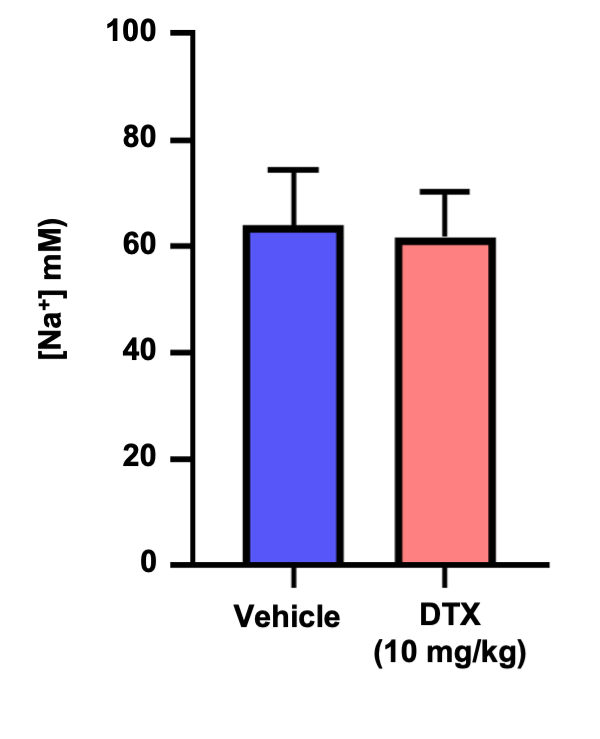

Supplement: Supplementary file 4 — Supplemental Figure 3 [file 41416_2022_1802_MOESM4_ESM.tif]

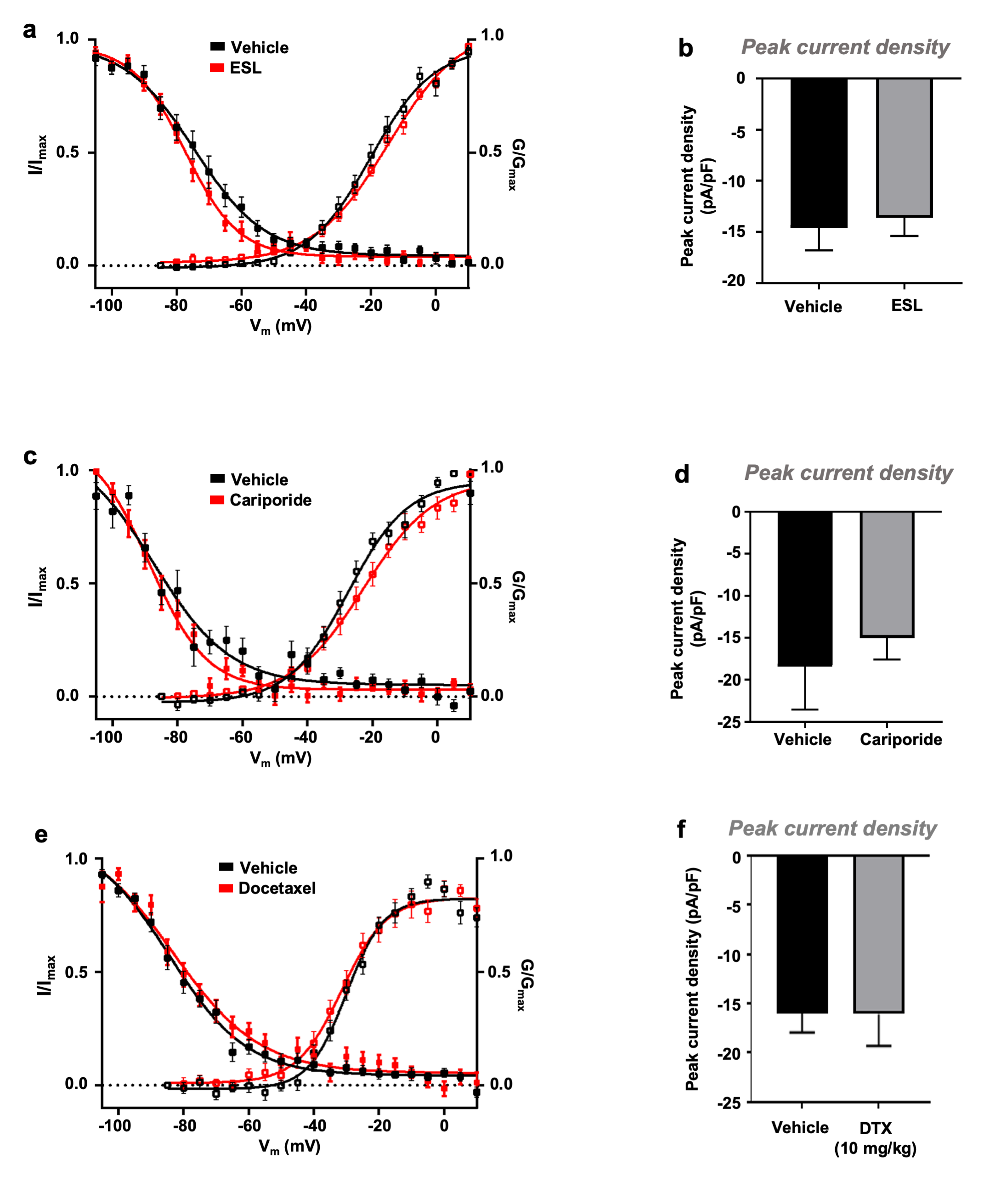

Supplement: Supplementary file 5 — Supplemental Figure 4 [file 41416_2022_1802_MOESM5_ESM.tif]
